# The administration of four-factor prothrombin complex concentrate exacerbates thrombin generation in trauma patients at risk of massive transfusion: an ancillary study of the PROCOAG trial

**DOI:** 10.1186/s13054-024-04836-z

**Published:** 2024-02-19

**Authors:** Jules Greze, Raphael Marlu, Mariette Baud, Landry Seyve, Tobias Gauss, Pierre Bouzat

**Affiliations:** 1 Inserm, U1216, CHU Grenoble Alpes, Grenoble Institute Neurosciences, Univ. Grenoble Alpes, 38000 Grenoble, France; 2 CNRS UMR5525 TIMC, Hemostasis Laboratory, CHU Grenoble Alpes, Univ. Grenoble Alpes, 38000 Grenoble, France; 3 Hemostasis Laboratory, CHU Grenoble Alpes, 38000 Grenoble, France; 4 Pôle d’Anesthésie-Réanimation, Hôpital Albert Michallon, BP 217, 38043 Grenoble, France

The use of four-factor prothrombin complex (4F-PCC) concentrate has been explored in the management of posttraumatic coagulopathy with the purpose of boosting thrombin generation [1]. The recent PROCOAG trial demonstrated no clinical benefit in patients at risk of massive transfusion after empiric administration of 4F-PCC, but an increased incidence of thromboembolic events in the 4F-PCC group compared to the placebo group [2]. To better understand the underlying coagulation mechanisms observed in this trial such as thrombin generation and modification of fibrinolysis, this PROCOAG ancillary study explored these pathways in a small sample of patients.

From 26th of March 2018 to 31st of August 2021, a subsample of adult trauma patients at risk of massive transfusion (Assessment of Blood Consumption score [3] ≥ 2 or clinical gestalt) from the PROCOAG trial were included at Grenoble University Hospital. These patients were randomized to receive either 4F-PCC (25UI of F IX/kg) or placebo (saline 0.9% 1 ml/kg) on admission. A specific Institutional Review Board approval and informed consent were obtained for this ancillary study (*Comité de Protection des Personnes Sud-Ouest et Outre-Mer 2, Toulouse, France,* 26th of March 2018). Samples were taken at H0 and H6 after admission to the resuscitation room to assess thrombin generation (TG) and fibrinolysis (see Additional file 1 for a detailed description of blood sampling techniques and assays). Statistical analyses were performed with GraphPad Prism 5.0 software using Wilcoxon matched-pairs signed-rank tests to compare parameters at H6 with baseline values and using Mann–Whitney test to compare placebo group to 4-F PCC group. Two-sided significance tests were used throughout with a *p* value threshold of 0.05.

A total of 24 patients were included with eleven patients allocated to the 4F-PCC group and thirteen patients to the placebo group (Flowchart in Additional file 2: eFigure 1). Additional file 2: eTable 1 summarizes the clinical characteristics and laboratory parameters. The sample is very representative of patients from the PROCOAG study population with a high percentage of patients in hemorrhagic shock, coagulopathy and a high traumatic load. More patients had at least one thromboembolic event in the 4F-PCC group compared to the placebo group: four patients versus two patients, respectively. The endogenous thrombin potential (ETP) and the thrombin peak were comparable at baseline in the two groups (for thrombin generation curves see Additional file 2: eFigure 2). After six hours, ETP and thrombin peak were significantly higher in the 4F-PCC group compared to the placebo group both with or without thrombomodulin (see Fig. 1A). Thrombomodulin-mediated inhibition of thrombin generation remained similar between the two groups (Additional file 2: eFigure 3). Assessments of fibrinolytic markers did not show major differences between the two groups: the median levels of plasmin-alpha2-antiplasmin complexes (PAP) and D-Dimers, reflecting the activation of fibrinolysis were similarly elevated in both groups (Fig. 1B). Furthermore, there were no difference in terms of levels of the main activator (t-PA) and inhibitors (alpha2-antiplasmin, PAI-1 and TAFIa/ai) between both groups (results available in Additional file 2: eFigure 4).

**Fig. 1:**
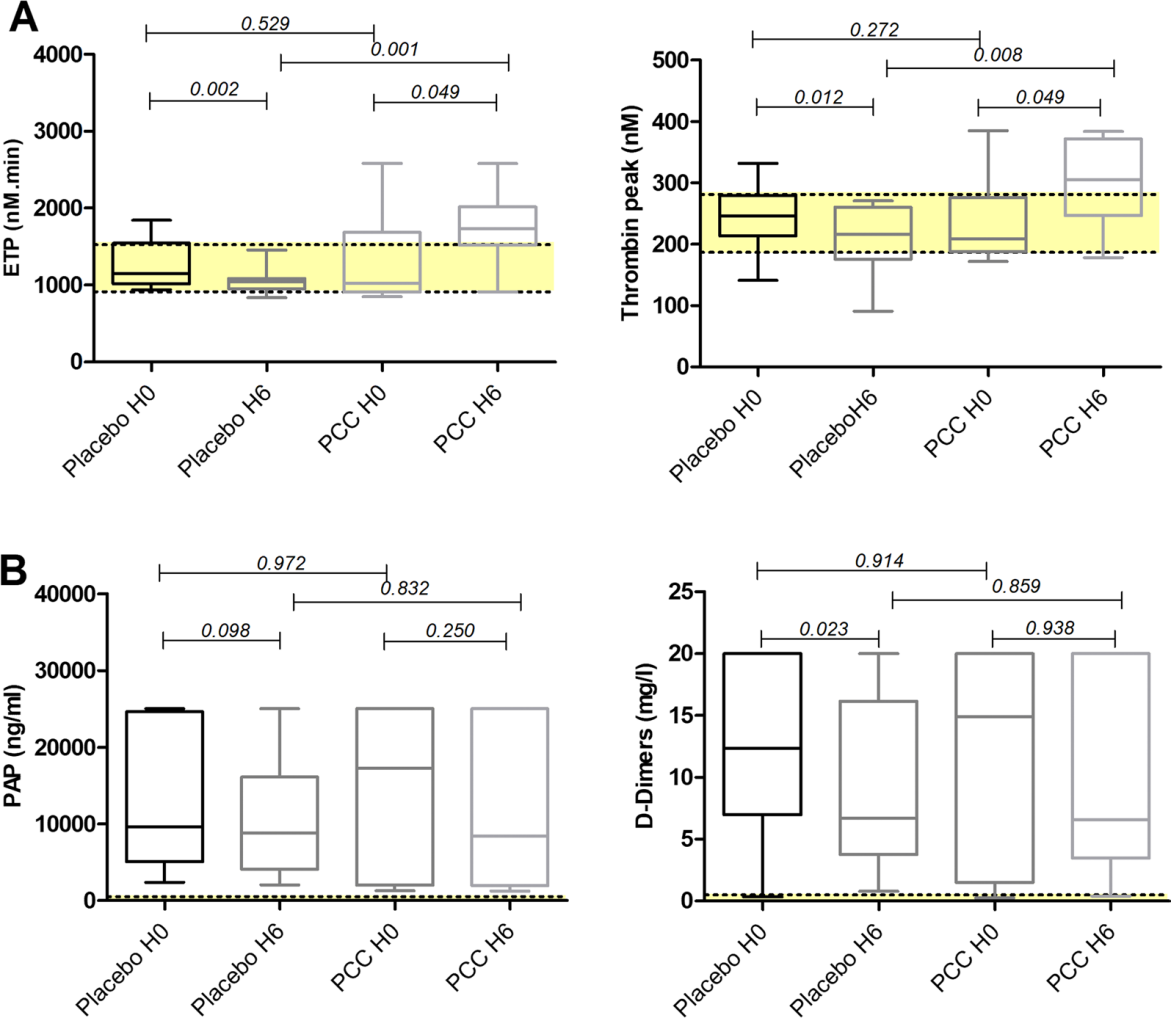
Thrombin generation and fibrinolysis assays. **A** Thrombin generation assays. Endogenous thrombin potential (ETP) and thrombin peak at H0 and H6 after hospital admission in the placebo group and in the four-factor prothrombin complex concentrate (4F-PCC) group. The yellow zone represents normal values. **B** Fibrinolysis assays. Plasmin-alpha2-antiplasmin (PAP) complex and D-Dimers at H0 and H6 after hospital admission in the placebo group and in the four-factor prothrombin complex concentrate (4F-PCC) group. The yellow zone represents normal values

This ancillary study from the PROCOAG trial indicates an increased thrombin generation at six hours after 4F-PCC administration. At baseline, trauma patients at risk of massive transfusion did not show inhibition of thrombin generation by thrombomodulin. This result further confirms other report performed on admission in coagulopathic trauma patients and treatment with PCC had previously been shown to increase thrombin generation (ETP) for several days in trauma patients [4]. Despite the use of tranexamic acid, fibrinolysis was activated at the same level at H0 and H6 in both groups, which is in line with recent fibrinolysis analysis after trauma [5]. The results suggest that empiric administration of 4F-PCC in patients at risk of massive transfusion generates as expected a thrombin burst and a prothrombotic state. Interestingly, there was no exhaustion of thrombin generation capacity at baseline. Supraphysiological thrombin generation observed in this ancillary study of the PROCOAG trial may explain increased incidence of thrombotic events after empiric administration of 4F-PCC.

### Supplementary Information

**Additional file 1**: Study protocol with blood sampling and assays.

**Additional file 2**: **eTable 1**: Patient characteristics by treatment group. **eFigure 1**. Flowchart of the study. **eFigure 2**. Median thrombin generation curves of patients from placebo and PCC groups. **eFigure 3**. Thrombomodulin-mediated inhibition of thrombin generation. **eFigure 4**. Levels of fibrinolysis activator and inhibitors.

## Data Availability

Pierre Bouzat had full access to all the data in the study and takes responsibility for the integrity of the data and the accuracy of the data analysis.
