# Supplementary material for: The administration of four-factor prothrombin complex concentrate exacerbates thrombin generation in trauma patients at risk of massive transfusion: an ancillary study of the PROCOAG trial
Source: Crit Care. 2024 Feb 19;28:51. doi: 10.1186/s13054-024-04836-z (PMC10875840; doi:10.1186/s13054-024-04836-z)
Supplement: Supplementary file 1 — Additional file 1: Study protocol with blood sampling and assays. [file 13054_2024_4836_MOESM1_ESM.docx]

Supplement 1: Blood sampling and assays

***Blood sampling and plasma preparation***

For fibrinolysis assays and thrombin generation assay, venous blood samples were collected into vacutainer CTAD tubes (Becton Dickinson, Le Pont de Claix, France) containing 0.109 M trisodium citrate, theophylline, adenosine and dipyridamole. Plasma was prepared by double centrifugation at 2,500 *g* for 15 min, collected, quick-frozen, and then stored at −80°C until analysis. The plasma samples were then thawed at 37°C in a water bath for 5 min before performing the assays.

***Fibrinolysis assays***

Levels of D-Dimers, plasminogen and alpha2-antiplasmin were measured on a STA-R Max coagulometer (Stago, Asnières, France) respectively with STA-Liatest D-Dimer kit (Stago), STA-Stachrom Plasminogen kit (Stago) and STA- Stachrom antiplasmin kit (Stago). Plasmin-antiplasmin (PAP) complex was determined with the Technozym PAP complex ELISA kit (Cryopep, Montpellier, France). Tissue plasminogen activator (t-PA), Plasminogen activator Inhibitor-1 (PAI-1) and TAFIa/ai were determined with ELISA kits purchased from STAGO, respectively Asserachrom t-PA, Asserachrom PAI-1 and Asserachrom TAFIa/ai.

***Thrombin generation assay (TGA)***

Thrombin generation was assessed using the calibrated automated thrombogram assay (Thrombinoscope BV, Maastricht, Netherlands) on an automated fluorometer (Fluoroscan Ascent, ThermoLab Systems, Franklin, USA). Coagulation was triggered by 5 pM of tissue factor (TF) and 4 µM of pro-coagulant phospholipids (PL) (PPP Reagent^®^ (Stago)). TGA was performed without and with 1 nM of recombinant human thrombomodulin (Cryopep, Montpellier, France) The thrombomodulin concentration was chosen to halve the mean ETP in healthy subjects.

For the preparation of the tissue factor/phospholipids/thrombomodulin mixture, 250 µl of a solution of thrombomodulin at 24 nM were added into a vial of PPP Reagent^®^ reconstituted with 750 µL (instead of one mL) of distilled water to get a mixture solution at 30 pM tissue factor, 24 µM phospholipids and 6 nM thrombomodulin. All TGAs were run in triplicate in 96-well plates under standard conditions. Briefly, 80 µL of PPP (poor platelet plasma) was mixed with 20 µL of tissue-factor/phospholipids or with 20 µL of the tissue factor/phospholipids/thrombomodulin mixture. Plates were incubated for 10 min at 37°C in an automated fluorimeter (Fluoroscan Ascent, ThermoLab Systems) before adding the fluorogenic substrate (ZGGR-AMC) and calcium (FluCa Kit®, Stago, France).
