# Supplementary material for: The administration of four-factor prothrombin complex concentrate exacerbates thrombin generation in trauma patients at risk of massive transfusion: an ancillary study of the PROCOAG trial
Source: Crit Care. 2024 Feb 19;28:51. doi: 10.1186/s13054-024-04836-z (PMC10875840; doi:10.1186/s13054-024-04836-z)
Supplement: Supplementary file 2 — Additional file 2: eTable 1: Patient characteristics by treatment group. eFigure 1. Flowchart of the study. eFigure 2. Median thrombin generation curves of patients from placebo and PCC groups. eFigure 3. Thrombomodulin-mediated inhibition of thrombin generation. eFigure 4. Levels of fibrinolysis activator and inhibitors. [file 13054_2024_4836_MOESM2_ESM.docx]

**The administration of Four-factor Prothrombin Complex Concentrate exacerbates thrombin generation in trauma patients at risk of massive transfusion: an ancillary study of the PROCOAG trial.**

Jules Greze MD, Raphael Marlu MD, Mariette Baud MD, Landry Seyve PhD, Tobias Gauss MD, and Pierre Bouzat MD, PhD

**Supplemental table and figures**

**eTable 1**: Patient characteristics by treatment group

|  | **4F-PCC Group**  **(n=11)** | **Placebo Group**  **(n=13)** |
| --- | --- | --- |
| Age, median (IQR), years | 34 (29-61) | 36 (27-46) |
| Female sex, No. | 0 | 4 |
| Male sex, No. | 11 | 9 |
| Blunt trauma, No. | 10 | 10 |
| Penetrating trauma, No. | 1 | 3 |
| **Prehospital variables** | | |
| Heart rate, median (IQR), beats/min | 111 (99-140) *N=9* | 114 (98-120) |
| Systolic arterial blood pressure, median (IQR), mmHg | 110 (95-120) *N=10* | 80 (70-110) *N=11* |
| Glasgow coma score, median (IQR)^a^ | 15 (12-15) | 14 (14-15) |
| Tranexamic acid infused, No. | 7 | 11 |
| Intubated, No. | 1 | 3 |
| Time from injury to arrival in the trauma bay, median (IQR), min | 110 (80-140) *N=9* | 87 (63-125) *N=10* |
| **Variables on arrival in the trauma bay** | | |
| Heart rate, median (IQR), beats/min | 110 (85-138) | 110 (89-120) |
| Systolic arterial blood pressure, median (IQR), mmHg | 100 (63-120) | 92 (75-103) *N=12* |
| Assessment of Blood Consumption score, median (IQR)^b^ | 1 (0-2) *N=9* | 1.5 (1-2) *N=12* |
| Patients with Assessment of Blood Consumption score ≥ 2 | 8 | 7 |
| Time from arrival to beginning of treatment, median (IQR), min | 28 (20-60) *N=10* | 25 (16-48) |
| Hemoglobin (g/dL), median (IQR), normal range (12-17 g/dL) | 10.9 (9.3-12.3) *N=8* | 11.7 (10.5-12.8) *N=7* |
| Lactate (mmol/L), median (IQR), normal is less than 1 mmol/L | 4.1 (3.8-4.4) *N=2* | 4.2 (2.8-9.5) *N=7* |
| Platelet count (x 10^9^/L), median (IQR), normal range (150-450 x 10^9^/L) | 206 (152-253) *N=8* | 235 (133-281) *N=7* |
| Fibrinogen (g/L), median (IQR), normal range (2-4 g/L) | 1.7 (1-2.2) *N=10* | 1.65 (1.4-2.45) *N=12* |
| Fibrinogen ≤ 1.5g/L | 5 | 4 |
| PT ratio, median (IQR)^c^, normal range (0.8-1.2) | 1.58 (1.36-1.72) | 1.34 (1.26-1.57) |
| PT ratio> 1.2 | 10 | 10 |
| PT ratio > 1.5 | 6 | 4 |
| Thromboelastometry clotting time, median (IQR), sec^d^, normal range (31-63 sec) | 67 (64-82) | 73 (66-80) |
| Thromboelastometry clotting time ≥ 80 sec, No. (%) | 4 | 3 |
| Thromboelastometry maximum lysis, median (IQR),%^e^, normal range (0-15) | 6 (0-8) | 6 (3-10) |
| Thromboelastometry maximum clot firmness, median (IQR), mm^f^, normal range (52-72) | 56 (52-63) | 60 (57-66) |
| AIS Head > 2, No. ^g^ | 3 *N=10* | 3 *N=11* |
| ISS, median (IQR)^h^ | 46 (38-75) *N=10* | 34 (29-54) *N=11* |
| ISS ≥ 15, No. | 10 | 11 |
| Revised Trauma score, median (IQR)^i^ | 7.1 (6.6-7.6) | 7.1 (6.1-7.6) |
| **Resuscitation^j^** | | |
| Need for hemostasis control procedure (surgical or radiological), No. | 8 | 6 |
| Transfusion of at least 3 units of RBCs within the first hour, No. | 5 | 6 |
| Transfusion of 10 units of RBCs or more within the first 24 hours, No. | 2 | 2 |
| Fibrinogen concentrate treatment, No. | 8 | 7 |
| Total dose of fibrinogen concentrate, median (IQR), g | 3.8 (2.6-4.5) *N=8* | 4.5 (3-6) *N=7* |

Abbreviations: 4F-PCC: Four factor Prothrombin Complex Concentrate; AIS: Abbreviated Injury Score; FFP: Fresh Frozen Plasma; IQR: Interquartile Range; ISS: Injury Severity Score; PT: Prothrombin Time; RBC: Red Blood Cell; a. The Glasgow Coma Scale measures level of consciousness based on eye, verbal, and motor responses. Ranging from 3 to 15, higher numbers indicate greater disability; b. The assessment of blood consumption score uses pulse rate, systolic blood pressure, ultrasound, and mechanism of injury to predict need for massive transfusion. It ranges from 0 to 4 with higher scores indicating greater likelihood of requiring massive transfusion. Patients with a score of less than 2 were enrolled in the trial as physician overrides, which was defined as a score of less than 2 and attending physician determination that a massive transfusion was needed; c. Prothrombin time (PT) ratio is the ratio between the prothrombin time of the patient and the prothrombin time reference value of the laboratory. A PTr higher than 1.2 indicates post-traumatic coagulopathy and a PTr higher than 1.5 indicates severe post-traumatic coagulopathy; d. Thromboelastometry clotting time is the time before the initiation of the clot measured with thromboelastometry using activators of the extrinsic pathway (EXTEM). A time higher than 80 seconds indicates PTr higher than 1.2; e. MCF is the greatest vertical amplitude of the trace. It reflects the absolute strength of the fibrin and platelet clot. f. Maximum Lysis is a parameter that describes the degree of fibrinolysis relative to the Maximum Clot Firmness (EXTEM) achieved during the measurement (Percent reduction of clot firmness after MCF in relation to MCF). A Maximum Lysis <15% is considered normal. g. The Abbreviated Injury Scale Head assesses head injury on a scale of 0 to 6 with 0 indicating no injury and 6 a fatal injury; h. The Injury Severity Score represents an overall assessment of bodily injury calculated as the sum of squares of the highest injury scores for body parts. It ranges from 0 to 75 with higher scores indicating greater injury. A score >15 indicates major trauma; i. The score range was 0 to 7.8. A higher score is associated with better survival probability; j. Includes observations made post randomization.

**eFigure 1: Flowchart of the study**

19 with physicians who refused to include

13 included in the primary analysis

11 included in the primary analysis

1 withdrew consent

2 with no blood sample

1 with no blood sample

16 treated with placebo

12 treated with 4F-PCC

47 patients screened for inclusion

28 randomized

**eFigure 2: Median thrombin generation curves of patients from placebo and PCC groups.**





**eFigure 3. Thrombomodulin mediated inhibition of thrombin generation.** The yellow zone represents normal values.

**

**

**eFigure 4. Levels of fibrinolysis activator and inhibitors.** The yellow zone represents normal values.
